# Gender Composition of Invited Speakers and Session Chairs at American Society for Apheresis Annual Meetings Between 2019 and 2024

**DOI:** 10.1002/jca.70015

**Published:** 2025-03-12

**Authors:** Jeremy W. Jacobs, Elizabeth S. Allen, Laura D. Stephens, Brian D. Adkins, Jennifer S. Woo, Allison P. Wheeler, Deva Sharma, Yvette M. Miller, Garrett S. Booth

**Affiliations:** ^1^ Division of Transfusion Medicine, Department of Pathology, Microbiology & Immunology Vanderbilt University Medical Center Nashville Tennessee USA; ^2^ Department of Pathology University of California San Diego La Jolla California USA; ^3^ Department of Pathology University of Texas Southwestern Medical Center Dallas Texas USA; ^4^ Department of Pathology City of Hope National Medical Center Irvine California USA; ^5^ Division of Pediatric Hematology/Oncology & Division of Hematology and Oncology University of Washington School of Medicine Seattle Washington USA; ^6^ Washington Center for Bleeding Disorders University of Washington School of Medicine Seattle Washington USA; ^7^ Division of Hematology‐Oncology, Department of Medicine Vanderbilt University Medical Center Nashville Tennessee USA; ^8^ American Red Cross Washington DC USA

**Keywords:** academic medicine, apheresis, conference speakers, diversity, equity, inclusion, transfusion medicine

## Abstract

Disparities persist throughout medicine, including among conference speakership invitations. The National Institutes of Health have highlighted the importance of diversity at academic conferences. We assessed the gender composition of speakers at the American Society for Apheresis (ASFA) annual meeting. We assessed all session chairs and speakers at the annual ASFA meeting from 2019 to 2024. Two authors independently assessed individuals' genders. The primary outcome was the gender composition of all session chairs and speakers by position. Subset analyzes were performed to assess the gender composition of unique individuals (i.e., examining the total number of unique men and women, independent of the number of sessions at which they spoke) and by professional degree. 820 positions (665 speaker positions and 155 chair positions) were identified; women comprised significantly more positions than men [64.3%, 528/820 (95% CI 61.1%–67.6%) vs. 35.6% 292/820 (32.4%–38.9%); *p* < 0.0001]. 52.7% (432/820) of all session positions were held by physicians, with no significant difference in the gender composition [women 47.5%, 205/432 (42.8%–52.2%) vs. men 52.6%, 227/432 (47.8%–57.2%); *p* = 0.31]. When limited to unique physician individuals, women were significantly outnumbered by men [40.1%, 71/177 (33.2%–47.5%) vs. 59.9%, 106/177 (52.5%–66.8%); *p* = 0.01]. This analysis demonstrated mixed findings, with more women across all positions overall but significantly more men when limited to unique physicians. Diversity in conference positions begets a broader array of perspectives, knowledge, and expertise, and can aid in realizing greater diversity in related areas. Thus, academic conference diversity should be prioritized and thoughtfully pursued.

## 1 Introduction

The composition of the medical profession is becoming increasingly diverse, with women representing an increasing proportion of applicants, matriculants, and graduates at U.S. medical schools [1]. However, these shifts have not translated to equitable opportunities within the sphere of academic medicine. Gender disparities persist in publication authorship, journal editorial board positions, research funding, recognition awards, academic promotion, and conference speakership invitations [2, 3, 4, 5, 6, 7, 8, 9, 10, 11, 12, 13].

While disparities within academic scholarship and advancement are complex, multifactorial, and intertwined, gender representation among invited conference speakers need not be. Dr. Francis Collins, former director of the National Institutes of Health, underscored this point in 2019 when he announced that he would not participate in conferences in which “attention to inclusiveness is not evident in the agenda.” [14] “Too often,” he noted, “women and members of other groups underrepresented in science are conspicuously missing in the marquee speaking slots at scientific meetings and other high‐level conferences.”

In the years following Dr. Collins's announcement, numerous studies have examined the representation of women at academic conferences and meetings; the majority have observed significant underrepresentation with variable improvement over time [15, 16, 17, 18, 19, 20, 21]. Subsequently, recommendations were published across a variety of disciplines to ensure conference speaker diversity [22, 23, 24]. Among these recommended initial steps is forming a diverse conference programming committee, which has been shown to yield greater diversity among conference speakers [9, 25]. This may necessitate a dedicated program committee policy that actively seeks diverse committee members. Additionally, collecting demographic data on the speakers at a particular conference over several years could then inform subsequent policy development. A dedicated speaker policy should delineate the committee's goals for its members and audience when constructing the speaker program. Further considerations should focus on the conference setting, venue, and programs available for attendees. For instance, utilizing funds to provide options such as childcare support (vs an opening reception) may make it more conducive for individuals to attend and/or speak at conferences who may have competing responsibilities or barriers to participation (e.g., childcare). Thoughtful consideration of scheduled social events and travel support could also support attendance for more individuals.

Given the mounting realization of the importance of diverse speakers at conferences, as well as the recommendations outlined in various guidelines [26, 27, 28, 29], we aimed to assess the gender composition of speakers at the American Society for Apheresis (ASFA) annual meetings over a six‐year period. Our primary goal was to describe the proportions of women and men among all session positions. Our secondary goals were to assess the gender proportions among all unique individuals, by speaker and chair positions individually, and by professional degree/position.

## 2 Materials and Methods

We compiled a list of all session chairs and speakers (inclusive of panelists and facilitators) at the annual ASFA meeting over a six‐year period (2019–2024) from the publicly available conference programs online (Table 1) [30, 31, 32, 33, 34, 35]. This time period was chosen because it comprises all ASFA conferences that have occurred since the year that the NIH made its public announcement regarding the importance of conference diversity. All presentation sessions during the meeting and all virtual/pre‐recorded sessions were included. Pre‐conference sessions, poster presentations, and private, invitation‐only sessions (e.g., Board of Directors sessions) were excluded from analysis.

**TABLE 1:** Sites of the annual ASFA meetings (2019–2024).

Year	Annual meeting program dates	Location
2019	May 14–18	Portland, OR
2020	September 23–25	N/A (Virtual meeting)
2021	May 12–15	N/A (Virtual meeting)
2022	May 4–6	Philadelphia, PA
2023	April 26–28	Minneapolis, MN
2024	April 17–19	Las Vegas, NV

Abbreviation: N/A, not applicable.

We obtained the gender, terminal degree, professional degree (if available), and session role (chair vs. speaker) for all included session positions. A profession‐based analysis was performed, which included physicians (MD, DO, or MBBS); nurses (NP, CRNP, MSN, BSN, RN); medical technologists (MLS, CLS, MT, SBB); and apheresis technicians (AT), as listed in the conference programs. Individuals without an academic degree/professional certification listed in the conference program were excluded from this analysis (*n* = 14), while those with at least one academic degree/professional certification mentioned in the programs were assumed to have their credentials accurately represented. Gender was determined via previously described methods [2, 3, 4, 36]. Two authors independently assessed individuals' gender via pronouns or stated gender from online sources. If unavailable, photographs were used to determine perceived gender (i.e., what the individual's gender was perceived as by the authors rather than the individual's self‐reported gender). This perception is not necessarily based on specific metrics, but rather reflects the culmination of each author's experience, worldviews, and perspectives, which inherently influence their perception of an individual. If these sources were unavailable, a web‐based gender identification tool (Genderize.io) was used to predict an individual's gender based on first and last name [37, 38]. There was 100% concordance for gender coding.

Our primary outcome was the gender composition of all session positions (chairs and speakers combined). We focused on gender by position, as each session position represents an opportunity for a unique individual to speak (i.e., each session could theoretically be presented by a distinct individual). However, we also performed several secondary analyzes, including (1) the gender composition of all session positions held specifically by physicians; (2) the gender composition of all unique individuals (i.e., the total number of unique men and women who served as chairs and speakers at sessions, independent of the number of sessions at which they spoke); (3) the gender composition of all unique physician individuals (i.e., the total number of unique men physicians and women physicians who served as chairs and speakers at sessions, independent of the number of sessions at which they spoke); (4) a temporal analysis over the six‐year timeframe; and (5) the gender composition of only chairs and only speakers. Finally, we assessed the composition of the three primary nonphysician professions, specifically nurses, medical technologists, and apheresis technicians. In this study, a “unique individual” refers to a distinct person who was counted only once in the “individual” analyzes, irrespective of the number of sessions they presented.

All statistical analyzes were conducted with Prism version 10.3.0 (GraphPad Software, La Jolla, CA, USA). Binomial tests were used to compare the proportion of women and men. 95% confidence intervals (CIs) were calculated and *p*‐values < 0.05 were considered statistically significant.

## 3 Results

### 3.1 All Positions

A total of 820 session positions (665 speaker positions and 155 chair positions) were identified over the six‐year study timeframe at the annual ASFA meetings (Table 2). Among all positions, women held significantly more positions than men [64.4%, 528/820 (95% CI 61.1%–67.6%) vs. 35.6% 292/820 (32.4%–38.9%); *p* < 0.0001]. Similar findings were observed when limited to speaker positions only [61.5%, 409/665 (57.8%–65.1%) vs. 38.5%, 256/665 (34.9%–42.3%); *p* < 0.0001] and chair positions only [76.8%, 119/155 (69.5%–82.7%) vs. 23.2%, 36/155 (17.3%–30.5%); *p* < 0.0001].

**TABLE 2:** Gender composition of speakers at the annual ASFA meeting (2019–2024).

	Men, no. (%)	Men, 95% CI	Women, no. (%)	Women, 95% CI	*p*
All positions					
Total positions (speakers and chairs) (*n* = 820)	292 (35.6%)	32.4%–38.9%	528 (64.4%)	61.1%–67.6%	< 0.0001
Total speaker positions (*n* = 665)	256 (38.5%)	34.9%–42.3%	409 (61.5%)	57.8%–65.1%	< 0.0001
Total chair positions (*n* = 155)	36 (23.2%)	17.3%–30.5%	119 (76.8%)	69.5%–82.7%	< 0.0001
All unique individuals					
Total unique individuals (speakers and chairs) (*n* = 323)	132 (40.9%)	35.6%–46.3%	191 (59.1%)	53.7%–64.4%	0.001
Total unique speakers (*n* = 309)	133 (43.0%)	37.6%–48.6%	176 (57.0%)	51.4%–62.4%	0.02
Total unique chairs (*n* = 69)	18 (26.1%)	17.2%–37.5%	51 (73.9%)	62.5%–82.8%	< 0.0001
All positions held by physicians					
Total physician positions (speakers and chairs) (*n* = 432)	227 (52.6%)	47.8%–57.2%	205 (47.5%)	42.8%–52.2%	0.31
Total physician speaker positions (*n* = 359)	198 (55.2%)	50.0%–60.2%	161 (44.9%)	39.8%–50.0%	0.06
Total physician chair positions (=73)	29 (39.7%)	29.3%–51.2%	44 (60.3%)	48.8%–70.7%	0.08
All unique physician individuals					
Total unique physician individuals (speakers and chairs) (*n* = 177)	106 (59.9%)	52.5%–66.8%	71 (40.1%)	33.2%–47.5%	0.01
Total unique physician speakers (*n* = 171)	106 (62.0%)	54.5%–68.9%	65 (38.0%)	31.1%–45.5%	0.002
Total unique physician chairs (*n* = 38)	16 (42.1%)	27.9%–57.8%	22 (57.9%)	42.2%–72.2%	0.412

### 3.2 Positions Held by Physicians

Approximately 52.7% (432/820) of all session positions were held by physicians, including 54.0% (359/665) of speaker positions and 47.1% (73/155) of chair positions. There was no significant difference in the gender composition of all session positions held by physicians [women 47.5%, 205/432 (42.8%–52.2%) vs. men 52.6%, 227/432 (47.8%–57.2%); *p* = 0.31], nor was there a difference when analyzing physician speaker positions only [women 44.9%, 161/359 (39.8%–50.0%) vs. men 55.2%, 198/359 (50.0%–60.2%); *p* = 0.06] or physician chair positions only [women 60.3%, 44/73 (48.8%–70.7%) vs. men 39.7%, 29/73 (29.3%–51.2%); *p* = 0.08].

### 3.3 Total Unique Individuals

A total of 323 unique individuals participated in the six annual ASFA meeting sessions. Of these, 309 unique individuals served as speakers, and 69 unique individuals served as chairs.

Overall, significantly more unique individuals were women [59.1%, 191/323 (53.7%–64.4%) vs. 40.9%, 132/323 (35.6%–46.3%); *p* = 0.001]. Similar findings were observed when limiting the analysis to only unique individual speakers [57.0%, 176/309 (51.4%–62.4%) vs. 43.0%, 133/309 (37.6%–48.6%); *p* = 0.02] and to only unique individual chairs [73.9%, 51/69 (62.5%–82.8%) vs. 26.1%, 18/69 (17.2%–37.5%); *p* < 0.0001].

### 3.4 Total Unique Physicians

Approximately 54.8% (177/323) of all unique individuals in the sessions were physicians, with unique physicians comprising 55.3% (171/309) of unique speakers and 55.1% (38/69) of unique chairs.

Women were significantly outnumbered by men among unique physicians overall [40.1%, 71/177 (33.2%–47.5%) vs. 59.9%, 106/177 (52.5%–66.8%); *p* = 0.01] and among unique physician speakers [38.0%, 65/171 (31.1%–45.5%) vs. 62.0%, 106/171 (54.5%–68.9%); *p* = 0.002]. There was no difference in the gender composition of unique physician chairs [women 57.9%, 22/38 (42.2%–72.2%) vs. 42.1%, 16/38 (27.9%–57.8%); *p* = 0.41].

### 3.5 Temporal Analysis

**FIGURE 1:**
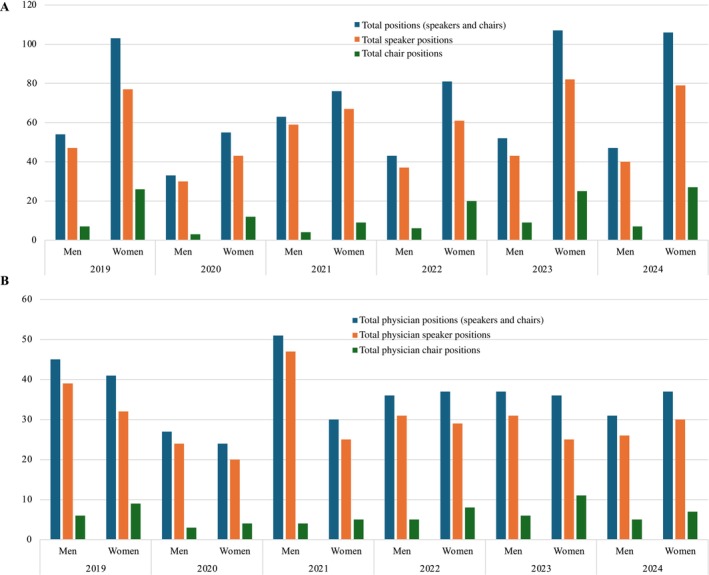
Gender composition of (A) all positions including speakers only and chairs only, and (B) positions held by physicians including speakers only and chairs only annually from 2019 to 2024.

**FIGURE 2:**
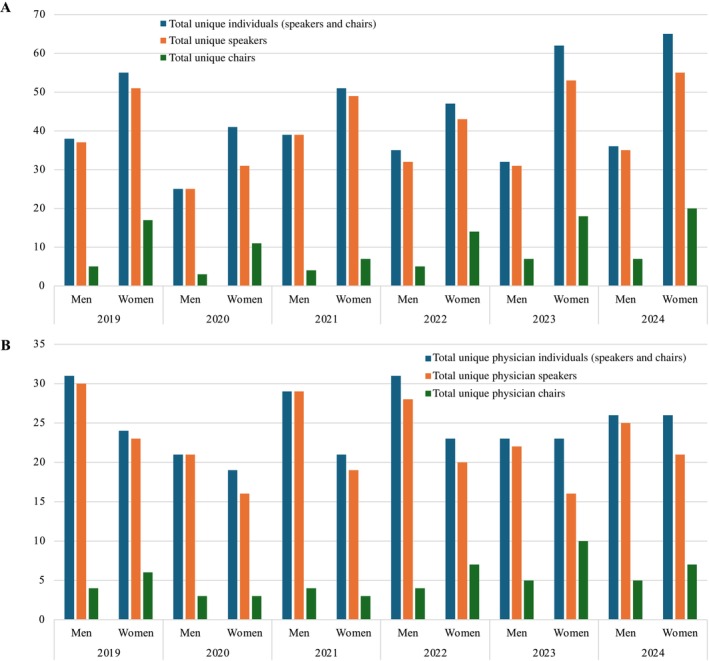
Gender composition of (A) unique individuals including speakers only and chairs only, and (B) unique physicians including speakers only and chairs only annually from 2019 to 2024.

**FIGURE 3:**
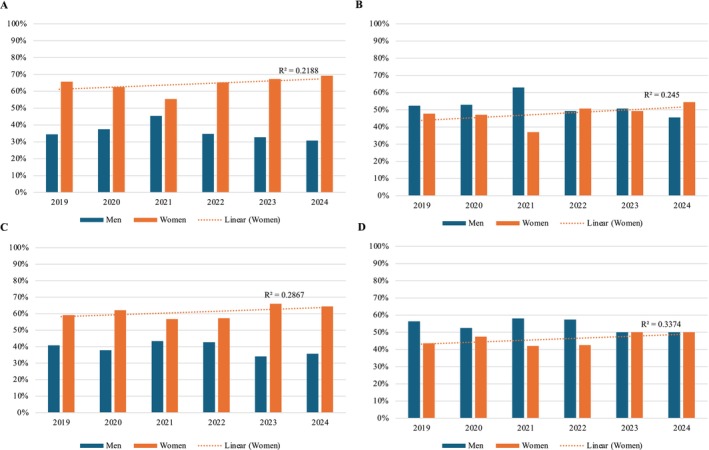
Temporal analysis of the proportion of women and men among (A) all positions, (B) physician positions, (C) unique individuals, (D) unique physicians. Trend line demonstrates the proportion of women annually.

The total number of annual session positions ranged from 88 (2020) to 159 (2023), with a median of 146 (IQR 128–155). The number of annual session positions held by physicians ranged from 51 (2020) to 86 (2019), with a median of 73 (IQR 69–79) (Figure 1). When limited to unique individuals, the total number of annual session positions ranged from 66 (2020) to 101 (2024), with a median of 91 (IQR 84–94); the number of unique physicians ranged from 40 (2020) to 54 (2022) with a median of 51 (IQR 47–54) (Figure 2). The proportion of women increased among all four groups (total positions, total unique individuals, total physician positions, and total unique physicians) between 2019 and 2024 (Figure 3).

### 3.6 Nurses, Medical Technologists, and Apheresis Technicians

Among total session positions, 16 were identified as apheresis technicians, 22 were identified as medical technologists/SBBs, and 277 had a nursing degree. Significantly more women than men were identified among both AT positions [100.0%, 16/16 (80.6%–100.0%) vs. 0.0%, 0/16 (0.0%–19.4%); *p* < 0.0001] and nursing positions [86.6%, 240/277 (82.1%–90.2%) vs. 13.4%, 37/277 (9.8%–17.9%); *p* < 0.0001]. Medical technologist positions were held by more men than women, but this was not statistically significant [68.2%, 15/22 (47.3%–83.6%) vs. 31.8%, 7/22 (16.4%–52.7%); *p* = 0.13]. When limited to unique individuals, there were two ATs (both women), 9 MTs/SBBs (6 women, 3 men), and 98 nurses (85 women, 13 men).

## 4 Discussion

In this analysis of the gender composition of speakers and chairs at the annual ASFA meetings from 2019 to 2024, women comprised significantly more session positions as speakers, chairs, and overall. In the secondary analysis of physicians only, there was no significant difference in the gender composition of annual meeting session positions. When evaluating unique individuals irrespective of professional degree, women significantly outnumbered men as speakers, chairs, and overall. However, when we analyzed only unique individual physicians, men significantly outnumbered women overall and as speakers, with no gender difference for unique physician chairs.

Overall, these results illustrate that almost two‐thirds of all sessions at the most recent annual ASFA meetings have been presented by women. Physicians spoke at approximately half of all sessions, without a significant difference in the gender composition. When evaluating unique individuals, women comprised more unique speakers overall, but more men physicians than women physicians served as speakers.

In performing these analyzes, it is important to distinguish between equity and parity. In this context, the former refers to the gender composition of a sample that approximates the overall population from which that sample may be drawn (e.g., the gender composition of physician speakers at ASFA compared to the overall gender composition of physicians who provide apheresis services). In contrast, parity is an equal 50–50 balance in gender composition. Whether equity or parity should be the goal is often debated. For fields dominated by a particular gender (e.g., orthopedic surgery, obstetrics and gynecology), parity may be a more appropriate target to ensure that a diverse range of experiences and viewpoints is available. If equity were sought instead in these instances, the composition of speakers would be significantly skewed to one gender, given that certain medical specialties are themselves skewed to that gender.

Given these considerations, our findings should be contextualized. We found that more unique men physicians were invited as speakers compared to women physicians. However, men comprise a greater proportion of the overall US physician workforce [39], including in non‐pediatric specialties that are frequently involved in apheresis (e.g., pathology, anesthesiology, critical care medicine, hematology, and nephrology) [40]. Similarly, significantly more women are registered nurses, and significantly more women nurses were invited to speak compared to men [39]. Thus, these findings demonstrate that much of the ASFA meeting speaker and chair positions are equitable. Conversely, workforce data have demonstrated that more than two‐thirds of medical technologists in the US are women [41, 42]; thus, our finding that, while not statistically significant, more than two‐thirds of positions occupied by medical technologists at the ASFA meetings were men suggests that opportunities may be less equitable for certain professions. Ultimately, though, whether equity or parity is the optimum goal in apheresis medicine remains uncertain.

Irrespective of the ideal target, the proportion of women entering medicine, including pathology and transfusion medicine, continues to increase. Notably, the 2021 Association for the Advancement of Blood and Biotherapies (AABB) Diversity, Equity, Inclusion, and Accessibility (DEIA) Survey, which was administered to active members and recently lapsed members of AABB in the USA and Canada, reported that 69% of respondents identified as women while 27% identified as men [43]. A similar initiative to characterize the demographics of ASFA would be informative. A conference that reflects the diversity of both society members and meeting participants benefits attendees through the acquisition of medical and scientific knowledge from a group with diverse experiences and expertise. It also benefits the presenters, as these roles are often viewed favorably for academic rank promotion and can result in collaborations and opportunities for grantsmanship and mentorship. Therefore, as the field of medicine diversifies, approaches to ensure diversity at scientific conferences must be prioritized. These may include developing a speaker policy with consideration of diversity; facilitating “family friendly” accommodations for caregivers; establishing a mentorship program and/or pairing senior women with early‐career or first‐time attendees; incentivizing participation in DEIA programs; and providing travel support in the form of grants or scholarships [22, 44].

This study has some limitations. We were only able to include those individuals with their degrees listed in the profession analysis. Further, we were unable to obtain statistics regarding the gender composition of ASFA members. However, as ASFA membership is not a prerequisite for speaking at the annual ASFA meeting, we do not believe this limits the veracity of our data. Additionally, we relied on online sources for gender determination instead of self‐identified gender, which may have hindered our ability to account for the full gender identity spectrum.

Importantly, we did not analyze other aspects of diversity such as age, race, ethnicity, country of origin, etc. Collecting these data is often not feasible and/or the methods are debated. In particular, race would be of interest for this analysis; however, self‐report is often invoked as the gold standard, and perceived race by observers can be questioned or may be unreliable. Nevertheless, a person's perceived identity (e.g., gender, race, ethnicity) is important, as implicit biases may be based on this perception irrespective of the person's self‐reported identity. We were unable to undertake these analyzes but emphasize that all aspects of diversity are important and should be systematically assessed. As such, we recommend that ASFA and other organizations systematically collect these data to facilitate future assessments of conference speaker diversity.

This analysis of the gender composition of ASFA annual meeting presenting speakers and session chairs yielded mixed findings. On the surface, the findings demonstrate gender diversity overall, with just over half of the positions occupied by women. However, analysis by profession shows that conference speakers followed the stereotypical gender norms: among nurses, the majority of speakers were women, and among physicians, the majority were men. It is because of the preponderance of nurses among conference positions that the overall balance tilted toward women. The inclusion of speakers and chairs from a diverse array of professions, including technicians, technologists, nurses, and physicians, is applauded, though technologists and technicians comprised a small minority of all positions, demonstrating an area of opportunity for improved representation. Further, within these groups, greater gender diversity should be prioritized by actively seeking diverse candidates. Although this analysis was not designed to assess causality, it is notable that there were more unique men as speakers, whereas more women were “repeat” speakers. Possible explanations are that fewer women have the professional connections or “celebrity status” to be invited, or perhaps they are less likely to step forward to speak at or chair sessions. Moreover, many organizations no longer provide funding for junior‐level staff to attend conferences. Senior‐level/tenured staff may have a greater ability to take time off to attend conferences and may have greater access to funding for travel. At present, a higher proportion of senior/tenured staff in the US are White men. Thus, the provision of funding and support at work for more junior individuals to attend and present at conferences would be beneficial with regard to diversity. Ultimately, we urge ASFA to continue to record, monitor, and set goals for gender diversity in this arena. Additionally, we recommend the organization collect self‐reported data regarding other demographic parameters to facilitate future analyzes of diversity.

Conference speaker and chair positions are one piece of the complex web of achievements that supports a career in academic medicine. Greater diversity in conference positions begets a broader array of perspectives, knowledge, and expertise at the conference podium and can aid in realizing greater diversity in related areas, such as publications, awards, and academic advancement.

## Disclosure

Allison P. Wheeler receives research funding from Octapharma, as well as honorarium for advisory board/consultancy from Bayer, BioMarin, CSL Behring, Genentech, Novo Nordisk, Octapharma, Pfizer, Sanofi, and Takeda. Garrett S. Booth has no disclosures related to this study; unrelated, he reports compensation for serving as a faculty speaker for a nonaccredited continuing education program and consulting for Grifols Diagnostics. All other authors report no relevant disclosures. Yvette M. Miller has no disclosures related to this study; received an award and honorarium from the Association for the Advancement of Blood and Biotherapies.

## Ethics Statement

The authors have nothing to report.

## Consent

The authors have nothing to report.

## Conflicts of Interest

The authors declare no conflicts of interest.

## Data Availability

The data that support the findings of this study are available from the corresponding author upon reasonable request.
